# Pathological Changes in Pulmonary Circulation in Carbon Tetrachloride (ccl_4_)-Induced Cirrhotic Mice

**DOI:** 10.1371/journal.pone.0096043

**Published:** 2014-04-24

**Authors:** Mita Das, Marjan Boerma, Jessica R. Goree, Elise G. Lavoie, Michel Fausther, Igor B. Gubrij, Amanda K. Pangle, Larry G. Johnson, Jonathan A. Dranoff

**Affiliations:** 1 Department of Internal Medicine, Division of Gastroenterology and Hepatology, University of Arkansas for Medical Sciences, Little Rock, Arkansas, United States of America; 2 Division of Pulmonary and Critical Care University of Arkansas for Medical Sciences, Little Rock, Arkansas, United States of America; 3 Department of Pharmaceutical Sciences, Division of Radiation Health, University of Arkansas for Medical Sciences, Little Rock, Arkansas, United States of America; 4 Research Service, Central Arkansas Veterans Health Service, Little Rock, Arkansas, United States of America; Vanderbilt University Medical Center, United States of America

## Abstract

**Rationale:**

Lack of an experimental model of portopulmonary hypertension (POPH) has been a major obstacle in understanding of pathophysiological mechanisms underlying the disease.

**Objective:**

We investigated the effects of CCl_4_-mediated cirrhosis on the pulmonary vasculature, as an initial step towards an improved understanding of POPH.

**Methods And Results:**

Male C57BL/6 mice received intraperitoneal injection of either sterile olive oil or CCl_4_ 3 times/week for 12 weeks. Cirrhosis and portal hypertension were confirmed by evidence of bridging fibrosis and nodule formation in CCl_4_-treated liver determined by trichrome/picrosirius red staining and an increase in spleen weight/body weight ratio, respectively. Staining for the oxidative stress marker, 4-hydroxynonenal (4-HNE), was strong in the liver but was absent in the lung, suggesting that CCl_4_ did not directly induce oxidative injury in the lung. Pulmonary acceleration time (PAT) and the ratio of PAT/pulmonary ejection time (PET) measured by echocardiography were significantly decreased in cirrhotic mice. Increase in right ventricle (RV) weight/body weight as well as in the weight ratio of RV/(left ventricle + septum) further demonstrated the presence of pathological changes in the pulmonary circulation in these mice. Histological examination revealed that lungs of cirrhotic mice have excessive accumulation of perivascular collagen and thickening of the media of the pulmonary artery.

**Conclusion:**

Collectively, our data demonstrate that chronic CCl_4_ treatment induces pathological changes in pulmonary circulation in cirrhotic mice. We propose that this murine cirrhotic model provides an exceptional tool for future studies of the molecular mechanisms mediating pulmonary vascular diseases associated with cirrhosis and for evaluation of novel therapeutic interventions.

## Introduction

Portopulmonary hypertension (POPH) is defined as pulmonary hypertension (PH) associated with portal hypertension, whether or not portal hypertension is secondary to underlying liver disease [1]. POPH can be a devastating complication in patients with advanced cirrhosis [2]. The added mortality patients experience due to POPH is clinically relevant [3]. One of the major concerns about POPH in cirrhotic patients is the variable improvement of PH in POPH undergoing liver transplantation; although some patients recover, others are left with life-threatening pulmonary hypertension despite normalization of liver function and portal hypertension [4].

Histopathological profiles of POPH are very similar to other forms of PH, including muscularization of small pulmonary arteries (PA), intimal thickening, smooth muscle hypertrophy, *in situ* thrombosis, plexiform lesions, and right ventricular (RV) hypertrophy [5]. Although deficiencies in endothelial prostacyclin synthase [6], excess circulating endothelin-1 [7], and PA platelet aggregates [8] have been documented in POPH patients, the molecular mechanisms driving the pathogenesis of the disease remain unclear. One of the primary reasons that the pathophysiological mechanisms directing POPH is still unknown is that animal, and in particular, mouse models of disease have not been developed.

To address this critical question, we tested the hypothesis that mice with cirrhosis induced by the widely used toxin, carbon tetrachloride (CCl_4_) [9], would show evidence of pathological alterations in pulmonary circulation. Here we report that CCl_4_-induced cirrhotic mice do indeed develop changes in pulmonary circulation that are similar to findings in human POPH. We believe that this experimental model will provide a highly useful model to test hypothetical mechanisms relevant to POPH by allowing the introduction of genetic, molecular, and biochemical approaches previously unavailable.

## Methods

### Ethics Statement

All animal studies were performed in accordance with the *Guide for the Care and Use of Laboratory Animals* of the National Institutes of Health and were approved by the Animal Care and Use Committee of the University of Arkansas for Medical Sciences (Protocol number 3398).

### Induction Of Cirrhosis By Ccl_4_ In Mice

Male C57BL/6 mice (age 6–8 weeks old, Charles River) were pre-treated with phenobarbital for one week (Baxter Health Corporation, Deerfield, IL) to up-regulate cytochrome P-450 enzymes [9],[10]. Phenobarbital was provided to mice in the drinking water (0.35 g/l) for 12 weeks. Mice (n = 7) received CCl_4_ mixed with sterile olive oil (1∶1, 0.4 ml/kg body weight) via intraperitoneal injection (IP) three times per week for 12 weeks. Control animals (n = 3) received olive oil only. Vitamin K (0.4 mg/kg body weight; Hospira, INC. Lake Forest, IL) was also administered to mice by subcutaneous injection to prevent bleeding throughout the study protocol.

### Echocardiography

Echocardiography of mice was performed in the University of Arkansas for Medical Sciences Biotelemetry & Ultrasound Imaging Core facility [11]. A Vevo 2100 high-resolution *in vivo* micro imaging system (VisualSonics, Toronto, Canada) with the MS400 Vevo MicroScan transducer (18–38 MHz) was used for ultrasound measurements. Animal anesthesia was induced with 2.5% isoflurane, and anesthesia was maintained with 1.5% isoflurane during imaging. All hair was removed from the chest with a depilatory cream. Short axis M-mode recordings at the mid left ventricular (LV) level were used to obtain conventional echocardiographic parameters, including the thickness of the left ventricular anterior wall, left ventricular posterior wall, left ventricular inner diameter, left ventricular volume, ejection fraction, fractional shortening, and stroke volume. Pulsed-wave Doppler was used to measure PA flow in short axis recordings at the level of the aortic valves. All analyses were performed with the Vevo 2100 cardiac analysis software package (VisualSonics).

### Histology And Immunohistochemistry

Mice were sacrificed by overdose of isoflurane after 12 weeks of CCl_4_ (experimental) or olive oil (control) treatment. Heart, lung, liver and spleen tissues were harvested. Pieces of liver tissue and right lung from each animal were placed in buffered 10% formalin solution overnight before embedding in paraffin. Paraffin-embedded liver and lung tissues were cut into 5 µm sections and stained with hematoxylin-eosin (H&E) to evaluate histology of the tissues. Trichrome and picrosirius red (PSR) stainings were performed on liver and lung sections according to the standard procedures to examine extracellular matrix and collagen accumulation, respectively. Stained liver and lung sections were scanned in the University of Arkansas for Medical Sciences Experimental Pathology Core facility with Aperio ScanScope CS2 using Imagescope software at 20× magnification.

### Spleen Index And Right Ventricular Hypertrophy

Isolated spleens from control and CCl_4_-treated mice were weighed and the spleen index (spleen weight/body weight) was calculated for evaluation of the signs of the development of portal hypertension [12].

To assess the degree of right ventricular hypertrophy, each isolated heart was dissected into the RV free wall and the left ventricle (LV) plus septum (S) and weighed separately. The ratio of RV to LV+S was calculated as an index of RV hypertrophy [13].

### Pulmonary Vascular Morphometry

Small pulmonary arterial wall thickness was measured by immunofluorescence staining of lung sections with antibody to α-smooth muscle actin (αSMA). Formalin-fixed and paraffin-embedded sections were deparaffinized in graded ethanol solutions. Antigenic sites of the lung tissue were unmasked by boiling in Na-citrate buffer (pH 5.5) using a pressure cooker. Immunofluorescence staining was performed with mouse anti-human αSMA primary antibody (clone 1A4, 1∶400; Sigma, St. Louis, MO) followed by Alexa594-conjugated goat anti-mouse IgG (1∶400; Invitrogen, Carlsbad, CA). Stained lung sections were scanned with Aperio ScanScope FL at 20× magnification. Wall thickness was assessed in αSMA positive staining in vessels (20–100 µm in diameter; n = 80–100 vessels) for each animal (n = 3 for each group). The wall thickness was measured using Aperio ImageScope software and calculated using the following equation:

Wall thickness  =  Wall width/Vessel width.

### Statistics

All data are expressed as arithmetic means ± SEM. Differences between groups were analyzed by either *t*-test or ANOVA followed by the Student-Newman-Keuls post-hoc test. Probability value <0.05 was considered significant.

## Results

### Long-Term Ccl_4_-Treatment Induces Liver Cirrhosis In Mice

**Figure 1:**
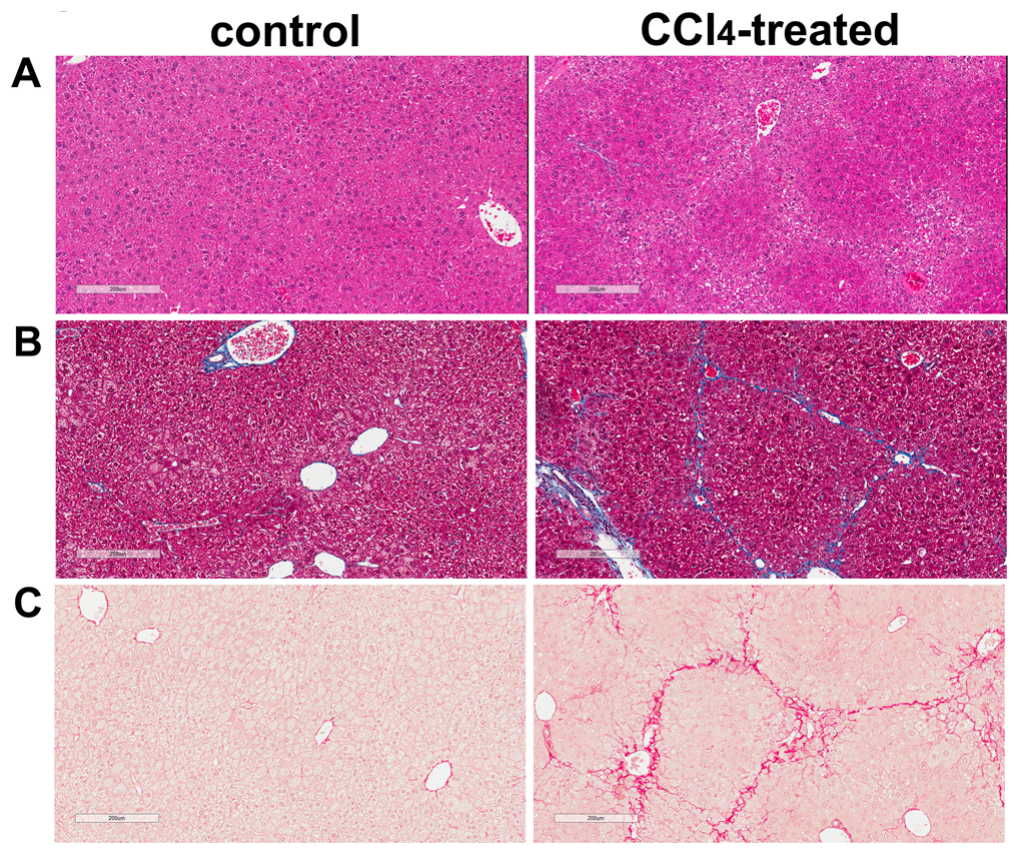
Long-term CCl_4_-treatment induces cirrhosis in mice. A. H&E-stained liver sections. Representative bright-field micrographs of H&E stained liver sections from mice injected three times per week for 12 weeks with olive oil only (control) and with CCl_4_ diluted in olive oil (CCl_4_). B. Trichrome-stained liver sections. Extracellular matrix deposition in liver was demonstrated using trichrome staining. C. PSR-stained liver sections. Collagen fibers were identified in liver by PSR staining. Scale bar  = 200 µm.

To induce cirrhosis, mice received IP injection of either CCl_4_ solution diluted with olive oil or olive oil alone three times per week for 12 weeks [9]. At the end of the experimental period, liver sections were stained with H&E, trichrome and PSR to evaluate histopathological changes and tissue fibrosis induced by CCl_4_ (Fig. 1). H&E staining of liver sections from all control mice revealed normal cellular architecture (Fig. 1A). In contrast, liver tissue from CCl_4_-treated group demonstrated mild steatosis and inflammation in the peri-central region. We then performed trichrome staining of the liver sections to assess extracellular matrix deposition (Fig. 1B). All control animals demonstrated a normal distribution of extracellular matrix, whereas CCl_4_-treated mouse livers demonstrated central-to-central bridging fibrosis and nodule formation. To further examine whether mice develop cirrhosis after prolonged CCl_4_-treatment, PSR staining was implemented on liver sections (Fig. 1C). The PSR staining patterns were similar to trichrome stains. Control mice had a normal distribution of collagen, whereas those treated with CCl_4_ demonstrated central-to-central bridging fibrosis and the presence of nodules. Collectively, these data confirm that long-term CCl_4_ administration (12 weeks) induces liver cirrhosis in mice in a fashion previously demonstrated [9], forming the basis for further experiments in this study.

### Ccl_4_-Induced Cirrhotic Mice Have Evidence Of Portal Hypertension

**Figure 2:**
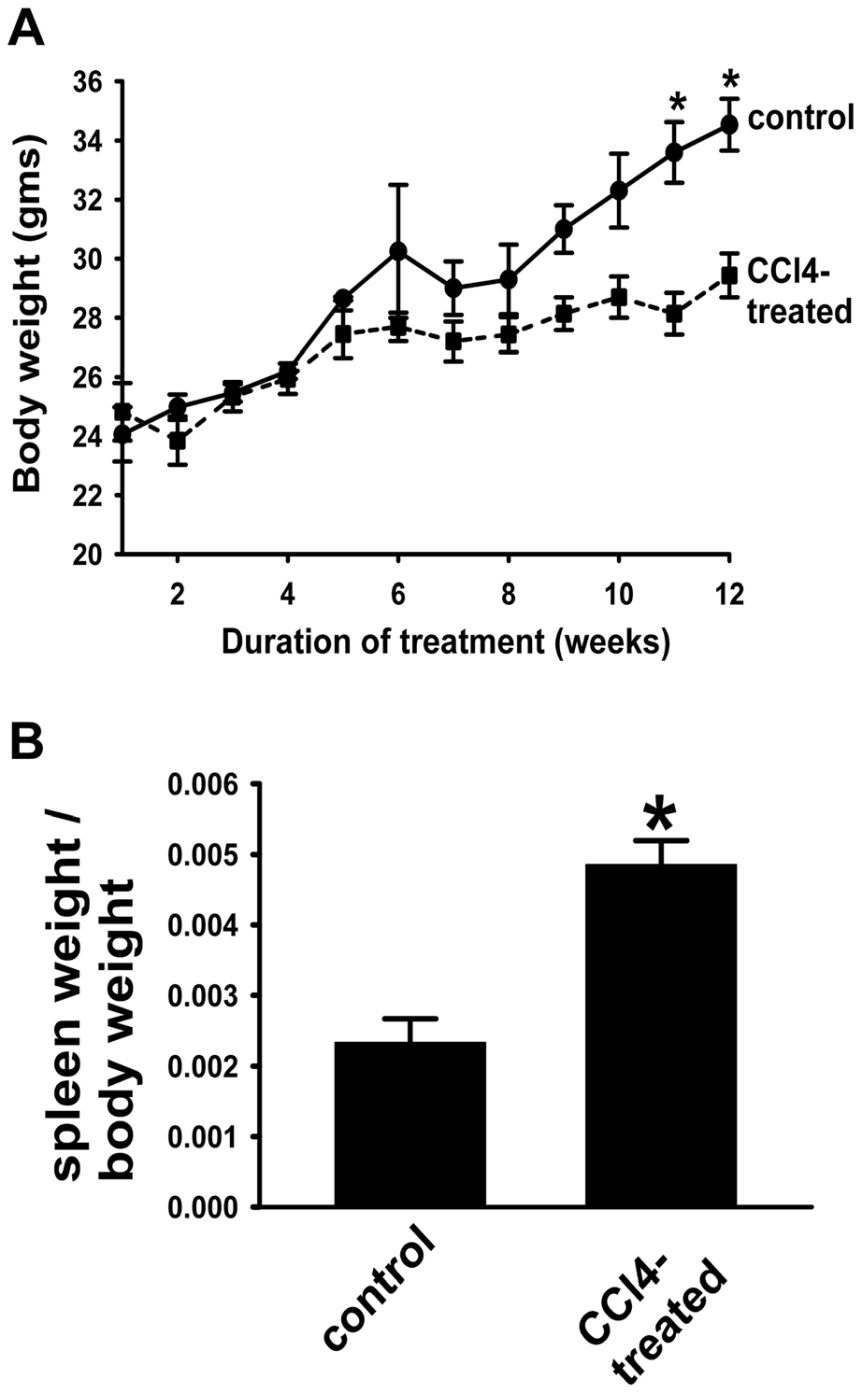
Spleen weight in mice is significantly increased by chronic CCl_4_ treatment. A. Total body weight. Total body weight of mice is significantly decreased by a 12 week-CCl_4_ treatment. *p<0.001, CCl_4_-treated *vs.* control animals. B. Spleen weight/body weight ratio. Increased ratio of spleen weight/total body weight in CCl_4_-treated mice is consistent with spleen enlargement. Data are mean ± SEM. *p<0.002, CCl_4_-treated *vs.* control mice. (n = 3 for control group; n = 7 for CCl_4_-treated group).

To evaluate whether prolonged CCl_4_ administration can induce the development of portal hypertension in cirrhotic animals, spleens from control and CCl_4_-treated animals were weighed. Chronic CCl_4_-treatment had marked effects on the total body weight of mice. Significant reduction in body weight was observed early after CCl_4_ administration and persisted throughout the entire study period (Fig. 2A), consistent with prior experimental observations [14]. In contrast, the spleen index (spleen weight/body weight) of the mice was significantly increased in CCl_4_-treated group compared to that of the control group (Fig. 2B), reflecting the development of splenomegaly due to portal hypertension (despite total animal weight loss) [14].

### The Oxidative Stress Marker 4-Hne Is Detected In The Liver, But Not Lung Of Ccl_4_-Treated Mice

**Figure 3:**
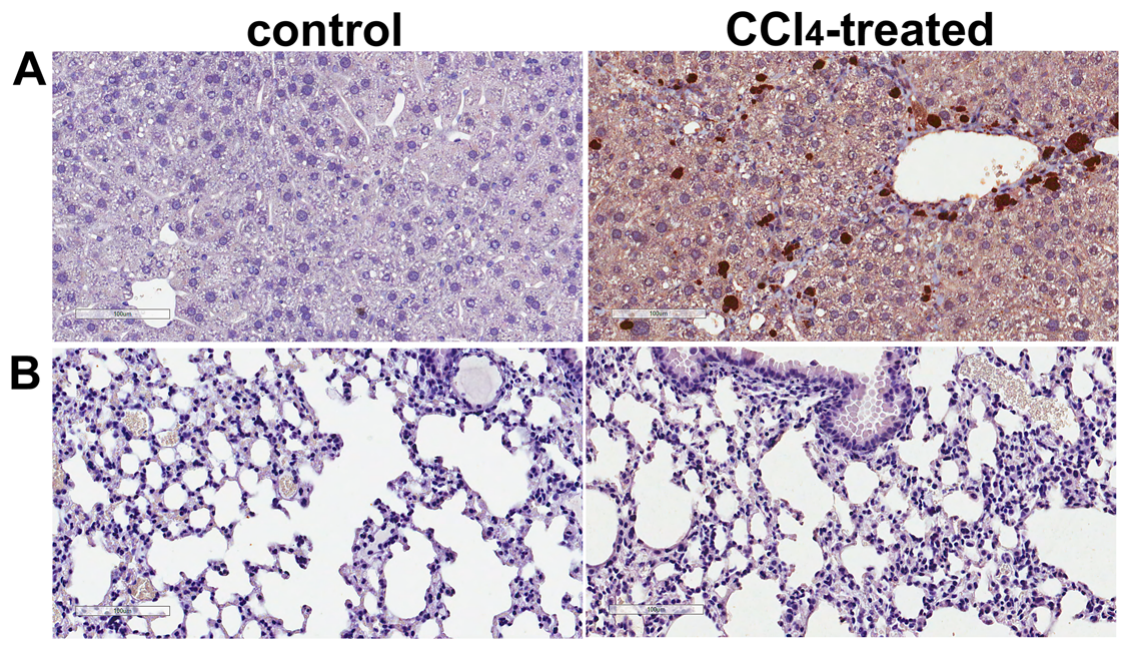
Oxidative stress evaluated by 4-hydroxynonenal (4-HNE) staining is selectively up-regulated in CCl_4_-treated liver. A. 4-HNE-stained liver sections. Representative bright-field photomicrographs of liver sections of control and CCl_4_-treated mice immunostained for 4-HNE. 4-HNE staining is evident in many lobular hepatocytes in CCl_4_-treated mice. B. 4-HNE-stained lung sections. Representative bright-field micrographs of 4-HNE-immunostained lung sections from control and CCl_4_-treated mice. In contradistinction to robust liver staining, no 4-HNE can be detected in the lungs of mice treated with CCl_4_. Scale bar  = 100 µm.

CCl_4_ induces liver injury primarily through oxidative stress. 4-HNE is a well-established marker of oxidative stress in tissues [15], so we examined 4-HNE in control and CCl_4_-treated liver and lung sections by immunohistochemistry. 4-HNE immunoreactivity was detected in hepatocytes within the peri-central region, lobule, and portal area of CCl_4_-treated liver, but not in control liver (Fig. 3A). To ensure that the changes noted in subsequent figures were not due to direct injury in lung in CCl_4_-treated animals, we similarly examined the lungs of control and experimental animals for the presence of 4-HNE (Fig. 3B). In contrast to Fig. 3A, no 4-HNE staining was detected in either control or CCl_4_-treated lungs, supporting the hypothesis that the lungs of CCl_4_-treated mice are not directly injured by mechanisms involving oxidative stress.

### Pulmonary Artery Acceleration Time (pat) Is Significantly Decreased In Cirrhotic Mice

**Figure 4:**
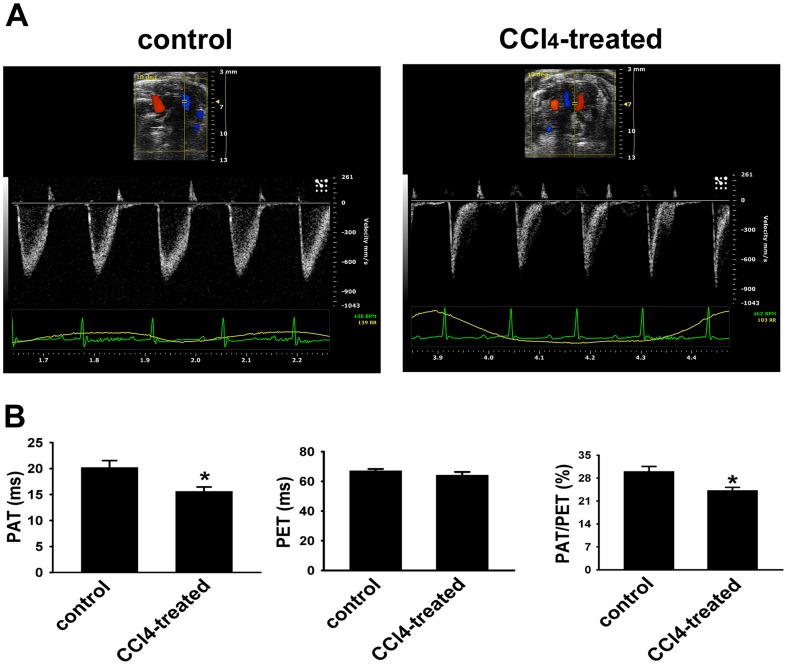
CCl_4_-treament induces reductions in pulmonary artery acceleration time (PAT) and the ratio of PAT/PET. A. Ultrasonographic images. Representative images of PA Doppler flow for control (left) and CCl_4_-treated (right) mice. Although the magnitude of the PET is identical, the PAT is markedly reduced in CCl_4_-treated mice. B. Quantification of PA hemodynamic parameters. Echocardiographic analysis was performed on control (n = 3) and CCl_4_-treated (n = 7) mice. Data are mean ± SEM per group. * *p*<0.01 CCl_4_-treated group *vs* control group.

Echocardiography was used to noninvasively assess the effects of CCl_4_-treatment on PA function. PAT (defined by the interval from the onset of RV ejection to peak flow velocity), as a surrogate of mean PA pressure [16], was measured by Pulsed Wave Doppler in control and CCl_4_-treated mice (Fig. 4A). Control animals showed characteristic gradual rise and fall of flow through the PA valve. After 12 weeks of CCl_4_ administration, such appearance of a distinctive PA flow was remarkably changed as shown in representative traces in Fig. 4A. We then quantified the observed changes in control and CCl_4_-treated mice (Fig. 4B). Reduction in PAT as well as in the ratio of PAT/PET was observed in cirrhotic mice compared with control animals. No difference in LV wall thickness or LV ejection fraction was observed in CCl_4_-treated mice (data not shown), suggesting that the alterations induced by CCl_4_ administration in mice were specific to right heart function. These data strongly suggest that chronic CCl_4_-treatment induced the development of alterations in pulmonary circulation in mice with portal hypertension.

### Long-Term Ccl_4_-Treatment Induces Rv Hypertrophy

**Figure 5:**
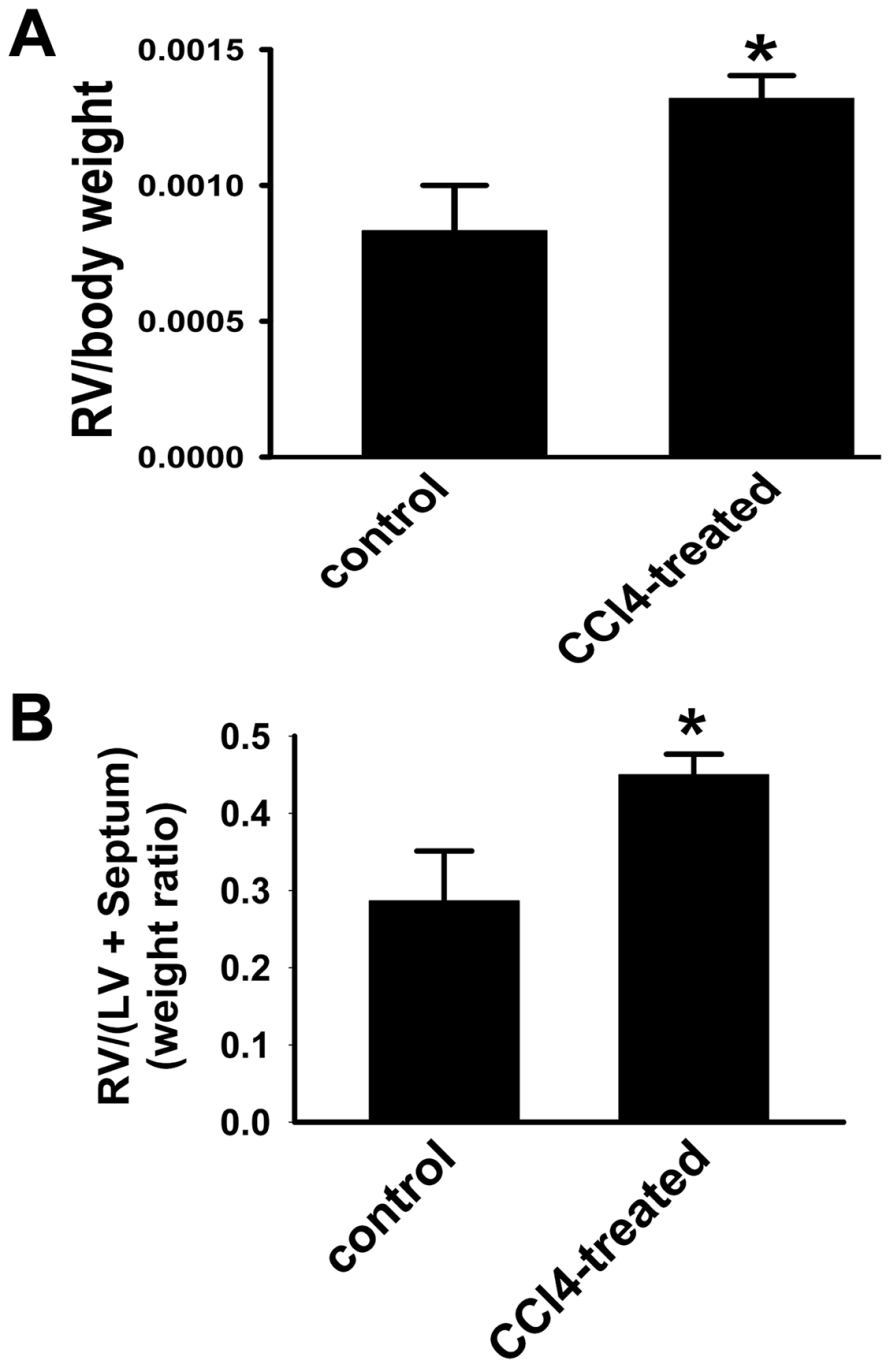
RV hypertrophy in mice occurs in response to chronic CCl_4_ treatment. A. Ratio of RV to total body weight. RV/total body weight ratio is increased in CCl_4_-treated mice. B. Ratio of RV to LV+S weight. RV/(LV+S) is augmented in CCl_4_-treated mice. Data are mean ± SEM. * *p*<0.02 CCl_4_-treated group *vs*. control group.

Pressure overload of the RV of the heart in response to PH induces RV hypertrophy, reflecting increased RV wall thickness and mass [13]. The ratios of RV weight relative to total body weight and RV mass relative to the sum of the (LV+septum) were therefore compared between control and CCl_4_-treated animals (Fig. 5). As predicted from the CCl_4_-induced modification of PA function, the results shown in Fig. 5A demonstrate that CCl_4_-treated mice exhibited significantly increased RV-to-body weight ratio than that of control animals. In contrast, LV-to-body weight ratios were equal for both groups (data not shown). RV mass relative to the (LV+septum) was also heightened in CCl_4_-treated mice (Fig. 5B). These physiological changes in the heart indicate that CCl_4_-treated mice develop significant RV hypertrophy, providing further evidence for the development of pathological changes in pulmonary circulation in cirrhotic mice.

### Marked Collagen Accumulation Occurs In The Lung Of Cirrhotic Mice

**Figure 6:**
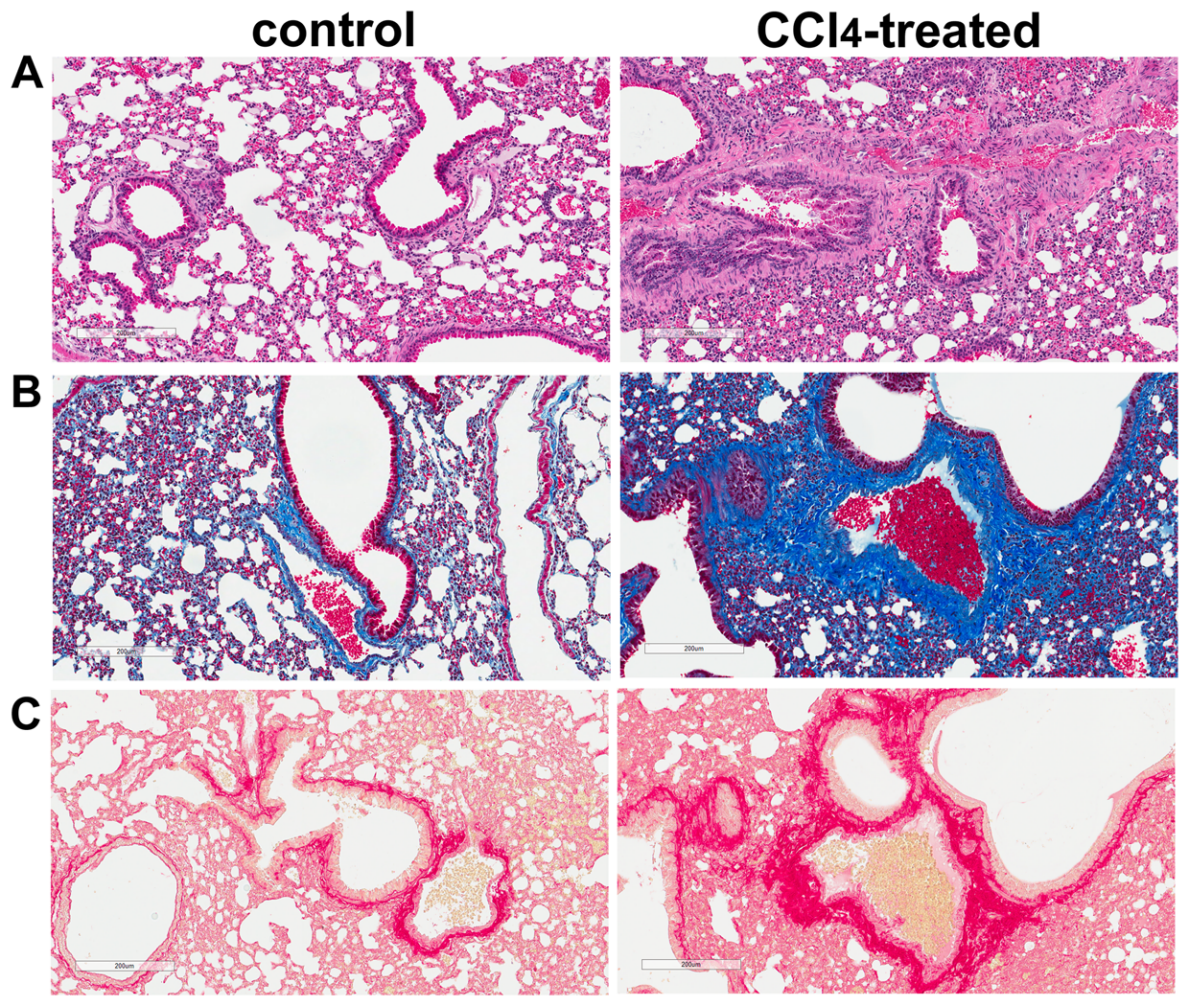
Marked collagen deposition is observed in lungs of the CCl_4_-treated mice. A. H&E-stained lung sections. Representative bright-field micrographs of H&E-stained lung sections from control and CCl_4_-treated mice. B. Trichrome-stained lung sections. Representative images of trichrome-stained lung sections identify increased extracellular matrix deposition in lungs of CCl_4_-treated mice. C. PSR-stained lung sections. Representative bright-field micrographs of PSR-stained lung sections illustrating increased collagen deposition in lungs of CCl_4_-treated mice. Scale bar  = 200 µm.

To evaluate the pathological changes in the lung of cirrhotic mice, lung sections from control and CCl_4_-treated animals were subjected to H&E, trichrome and PSR staining (Fig. 6). Cirrhotic mice exhibited severe pulmonary fibrosis, characterized by loss of normal alveolar architecture, prominent disorganized thickening of the alveolar septa, and collapse of the alveolar space, which was most evident on H&E staining (Fig. 6A). Trichrome staining of lung sections demonstrated intense blue staining, indicative of extracellular matrix deposition in the peribronchial and perivascular adventitia, of the lung of cirrhotic animals (Fig. 6B). Substantial accumulation of collagen, evaluated by PSR staining, was also observed in the vessel media and adventitia as well as in lung parenchyma of the CCl_4_-treated animals compared with the control groups (Fig. 6C). Taken together, these data strongly suggest the presence of noticeable fibrotic lesions in the lung of CCl_4_-induced cirrhotic mice.

### Vascular Wall Thickness In The Lung Of Cirrhotic Mice Is Significantly Increased

**Figure 7:**
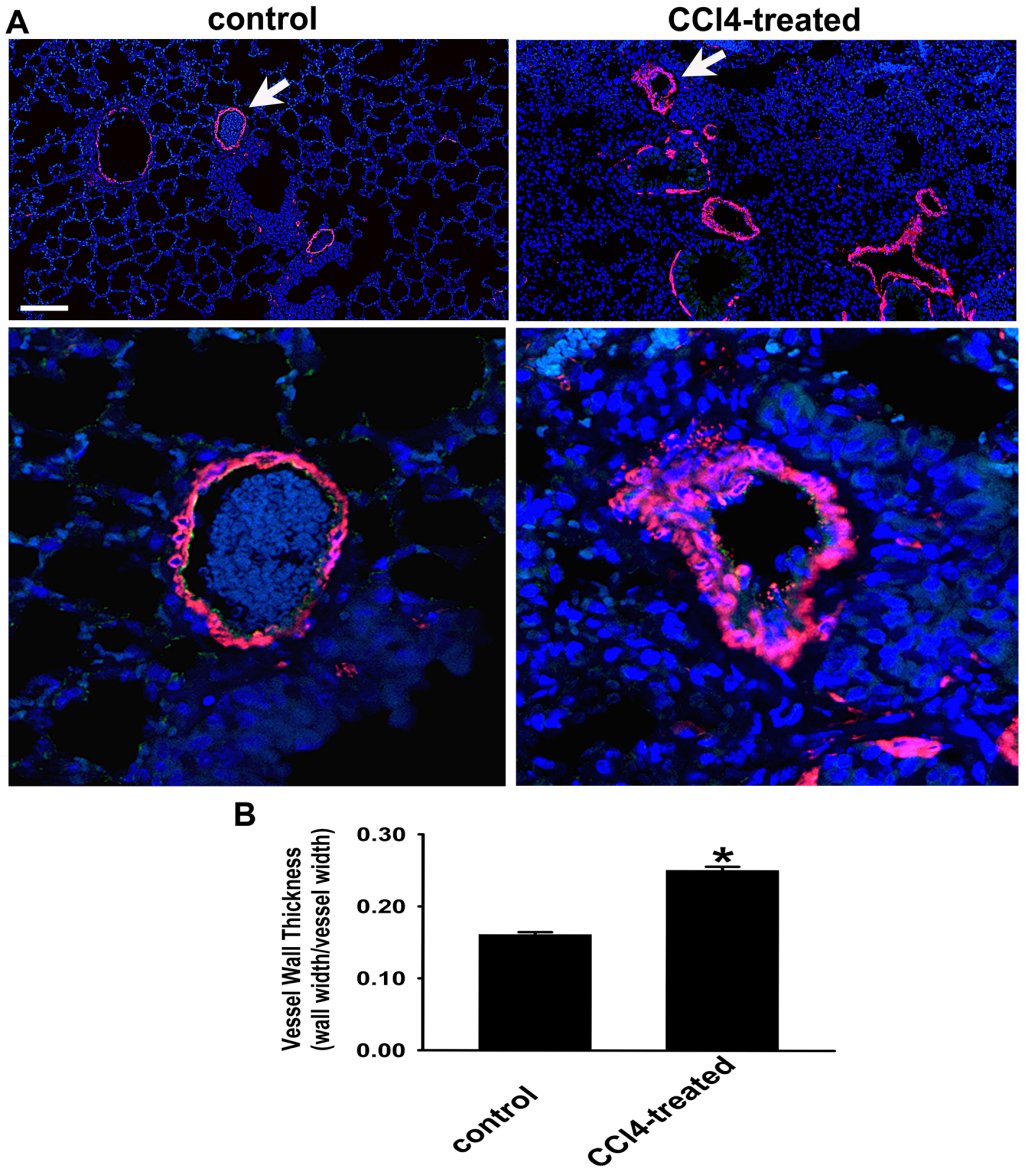
Lung vascular wall is significantly thickened by chronic CCl_4_-treatment. A. Immunofluorescent detection of αSMA. Identification of αSMA-positive (red color) vessels in lung sections from control and CCl_4_-treated mice. Nuclei are blue. Arrows represent magnified vessels from control and experimental animals in the lower panels. Scale bar  = 100 µm (upper panel) and 20 µm (lower panel). B. Quantification of arterial wall thickness. Wall thickness of arteries (20–100 µm in diameter) is markedly upregulated in lungs of CCl_4_-treated mice. n = 3 animals/group; 80–100 vessels were examined in each animal. * *p*<0.001 CCl_4_-treated group *vs* control group.

Since remodeling of small pulmonary arteries in the lung is the key pathological feature of PH [17], we then evaluated the vascular structure in the control and cirrhotic mice by immunofluorescent staining for αSMA. Examination of αSMA staining in the CCl_4_-treated mouse lungs revealed marked vascular medial wall remodeling compared to that in the control lung (Fig. 7A). Measurement of wall thickness of αSMA-positive vessels (20–100 µm diameter) also demonstrated significant increase in vessel wall thickness in the lungs of cirrhotic mice (Fig. 7B) providing yet another index consistent with development of the pathological changes in pulmonary circulation in these animals.

## Discussion

Here, we report development of the experimental animal model of pathological changes in pulmonary circulation associated with cirrhosis. The earliest description of the association between hepatic and lung vascular disorders was provided by Mantz and Craig in 1951, and since then a number of case reports and case series have validated this observation [18]. Despite such overwhelming evidence for the co-existence of the vascular injury in the liver and in the lung, to date, there are only few published reports describing experimental animal models of pulmonary vascular complications induced by cirrhosis that can be related to POPH [19],[20]. Quite surprisingly, demonstration of the murine model discussed herein did not require difficult experimental methods, exotic in vivo approaches, or rare mouse models. Rather, simple 12-week treatment with CCl_4_, which has long been known to induce cirrhosis and portal hypertension in rodents, was sufficient also to induce changes in the pulmonary circulation. Importantly this pathologic phenotype of cirrhotic mice characterized by liver/lung injury, displayed pronounced oxidative stress selectively in the liver but not in the lungs, suggesting that lung injury was not directly due to CCl_4_-induced oxidative damage. Use of a high-frequency ultrasound system in our studies allowed clearly defining spectral Doppler signals from the proximal PA in control and CCl_4_-treated mice. The Doppler waveforms showed marked shortening of the PAT and PAT/PET ratio in cirrhotic mice compared to the control group. RV hypertrophy and marked accumulation of collagen as well as thickening of small PA wall in the lungs of cirrhotic mice are consistent with findings in POPH patients [21]. Taken together, these data provided evidence that the lung vascular injury induced right heart strain, as would be expected from findings in POPH patients [22]. However, it is important to note that, in absence of direct right ventricular pressure measurements, it is premature to describe our current model as “mouse POPH”.

The pathogenesis of POPH is not completely understood, but likely involves a complex interaction of several mechanisms. Cirrhotic or noncirrhotic portal hypertension has been found to be a pre-requisite for the development of PH [23]. In the current study, liver pathology of CCl_4_-treated mice revealed the presence of bridging fibrosis and nodules defining the onset of cirrhosis. Earlier studies demonstrated that in cirrhotic mice increase in spleen weight positively correlates with portal hypertension [12]. Our study provides key additional evidence in support of the idea that chronic CCl_4_ treatment generates a cirrhotic mouse model of portal hypertension.

Profound hepatotoxicity of CCl_4_ and consequent hepatic cirrhosis require generation of reactive oxygen species (ROS) by the enzymatic complex of cytochrome P450 [15]. Such pathology is engendered by the metabolism of CCl_4_ that initiates peroxidation of polyunsaturated fatty acids producing α, β-unsaturated aldehydes, such as 4-HNE and malondialdehyde (MDA) [15]. Involvement of fatty acid peroxidation in hepatotoxicity was also found in our current study as we have observed a major increase in 4-HNE in the liver of cirrhotic mice. In contrast, 4-HNE was not detected in the lung of cirrhotic mice. The absence of 4-HNE in the lungs of our CCl_4_-treated mice is consistent with lack of lipid peroxidation, but is in contrast with studies in CCl_4_-treated rats that reported presence of lipid peroxidation in the lung as determined by the elevation of MDA in lungs with CCl_4_ treatment [19],[20],[24]. Species-specific differences in histopathological and molecular markers in the setting of chronic liver disease might be explained by differences in metabolism of ROS-generating toxins between mice and rats.

In the present study, we have used noninvasive transthoracic echocardiography to monitor the development of right heart strain in CCl_4_-induced cirrhotic mouse model. PAT measurement has been shown to correlate with invasively measured mean PA pressure [16]. Our observation that the exposure of mice to CCl_4_ for 12 weeks decreases PAT as well as PAT/PET ratio is consistent with the idea that CCl_4_ administration may induce an increase in PA pressure. Although proximal PA flow, as assessed by pulsed wave Doppler, has a characteristic pressure-dependent pattern, in which the acceleration phase becomes shorter as the PA pressure increases, additional factors, including heart rate, cardiac output, RV function, and nuances of the imaging technique, may affect its duration [25]. LV function and size (ejection fraction, stroke index, and LV mass) did not differ significantly between the two groups of animals, suggesting that effects of chronic CCl_4_-treatment are selectively limited to changes in the PA. In addition, the distinct echocardiographic patterns observed in CCl_4_-treated mice correlated to the RV hypertrophy in the cirrhotic mice. Taken together, these data strongly suggest that CCl_4_ initiates a pathogenic process in the pulmonary circulation of cirrhotic mice.

Hypertrophy of vascular smooth muscle in pulmonary arterioles and accumulation of extracellular matrix are the hallmarks of PH [26]. Histological analysis of the lungs in the cirrhotic animals presented here demonstrated structural remodeling characterized by an increased deposition of collagen in the media and adventitia of arterioles as well as in the parenchyma of the lung from the CCl_4_-treated group as compared with the control group. In addition, significant remodeling observed in the small PA within the lungs of cirrhotic mice predicts the pervasive nature of the pathological process initiated by CCl_4_. The above-mentioned pathohistological features provide additional evidence of the link between lung vascular changes and cirrhosis.

The limitations of this study are also worth noting. Specifically, this study is largely observational and thus does not provide direct insight into the cellular pathogenesis of POPH or related conditions. Frankly, we were surprised to find that evidence of such changes in CCl_4_-treated mice had not been reported previously, so we worked rigorously to define the extent of lung injury and right heart strain in these animals. We look forward to having our work replicated and advanced by other laboratories, as there is a critical need for basic research in cirrhosis-related lung disease, including POPH. Although the pathogenesis of POPH likely involves several converging mechanisms, the common themes that are relevant are a disordered liver-to-lung signaling axis and alterations in soluble mediators of vascular growth, remodeling, and thrombosis [5]. Use of our model will now allow investigators to test the relative importance of such pathways as deficiencies in endothelial prostacyclin synthase, excess circulating endothelin-1, and PA platelet aggregates [6]–[8].

In summary, we report that mice in which cirrhosis and portal hypertension is induced by long-term CCl_4_ treatment also develop pathological changes in pulmonary circulation with vascular lesions that might be analogous to human POPH to warrant use of this model in future pathophysiological studies. We sincerely hope that this will ultimately lead to novel, effective therapies to prevent or reverse POPH in affected patients.
